# Novel subtractive transcription-based amplification of mRNA (STAR) method and its application in search of rare and differentially expressed genes in AD brains

**DOI:** 10.1186/1471-2164-7-286

**Published:** 2006-11-07

**Authors:** Qing Yan Liu, Roy R Sooknanan, Lawrence T Malek, Maria Ribecco-Lutkiewicz, Joy X Lei, Hui Shen, Boleslaw Lach, P Roy Walker, Joel Martin, Marianna Sikorska

**Affiliations:** 1 Neurobiology Program, Institute for Biological Sciences, National Research Council of Canada, Ottawa, Ontario, K1A 0R6,Canada; 2 Alethia Biotheraputics Inc., 8475 Christophe-Colomb Avenue, Suite 1000 Montreal, Quebec, H2M 2N9, Canada; 3 Osteopharm Inc., Unit 14, 1155 North Service Road, Oakville, Ontario, L6M 3E3, Canada; 4 Hamilton Health Sciences, Hamilton General Hospital, Laboratory Medicine, 237 Barton Str East, Hamilton, Ontario, L8L-2X2, Canada; 5 Interactive Information Group, Institute for Information Technology, National Research Council of Canada, Ottawa, Ontario, K1A 0R6, Canada

## Abstract

**Background:**

Alzheimer's disease (AD) is a complex disorder that involves multiple biological processes. Many genes implicated in these processes may be present in low abundance in the human brain. DNA microarray analysis identifies changed genes that are expressed at high or moderate levels. Complementary to this approach, we described here a novel technology designed specifically to isolate rare and novel genes previously undetectable by other methods. We have used this method to identify differentially expressed genes in brains affected by AD. Our method, termed Subtractive Transcription-based Amplification of mRNA (STAR), is a combination of subtractive RNA/DNA hybridization and RNA amplification, which allows the removal of non-differentially expressed transcripts and the linear amplification of the differentially expressed genes.

**Results:**

Using the STAR technology we have identified over 800 differentially expressed sequences in AD brains, both up- and down- regulated, compared to age-matched controls. Over 55% of the sequences represent genes of unknown function and roughly half of them were novel and rare discoveries in the human brain. The expression changes of nearly 80 unique genes were further confirmed by qRT-PCR and the association of additional genes with AD and/or neurodegeneration was established using an in-house literature mining tool (LitMiner).

**Conclusion:**

The STAR process significantly amplifies unique and rare sequences relative to abundant housekeeping genes and, as a consequence, identifies genes not previously linked to AD. This method also offers new opportunities to study the subtle changes in gene expression that potentially contribute to the development and/or progression of AD.

## Background

Recent advance in molecular biology have introduced new high-throughput tools for the analysis of differential gene expression in complex diseases, such as Alzheimer (AD), providing simultaneous overviews of the genes or proteins associated with multiple cellular pathways. The most commonly used technology for the assessment of gene expression changes in postmortem brain is the DNA microarray [1-5] This approach has not only confirmed the involvement of genes implicated in AD by conventional methods, but also revealed changes in additional genes, not previously associated with AD [6,7]. However, as this method requires *a priori *knowledge of gene sequences, it cannot be applied as a discovery tool for novel transcripts. Furthermore, the expression levels of low abundance genes cannot be readily assessed by DNA microarray hybridization, since reliable results are usually obtained only for genes that are expressed in high or moderate levels. This is a significant limitation since many transcripts expressed preferentially in brain (e.g., neurotransmitter receptors and their regulatory factors) are present at very low levels [8,9].

Differential display and conventional subtractive hybridization approaches are capable of detecting expression changes in both known and novel genes. Differential display uses arbitrarily primed PCR to fingerprint differences (from first strand cDNA) in gene expression between two samples, with the results being determined by the intensities of bands on a polyacrylamide gel [10]. The major disadvantages of this method include its lack of sensitivity for the detection of rare RNA species, the high number of false positives generated during PCR and cloning of the differentially expressed products from low resolution polyacrylamide gels, where an apparent single band may contain multiple cDNA species. Consequently, differential display is labor intensive and unreliable for this application.

Subtractive hybridization, on the other hand, permits the isolation of target sequences from one single-stranded DNA population, referred to as "tester", from another DNA population, referred to as "driver" by using an excess of sequences. The two populations are mixed and put through iterative rounds of subtraction of cross-hybridized products. Earlier subtractive methods required physical removal of hybridized driver and tester sequences, which contributed to the loss of low abundance tester sequences. Suppression subtractive hybridization (SSH) is a newer method [11] which couples hybridization-based de-selection of common cDNAs to PCR amplification which enriches differentially expressed transcripts from two mRNA sources. In contrast to differential display, the primers for PCR amplification are clearly defined, thus avoiding problems associated with random primers. The main disadvantage of this procedure is its higher detection threshold. According to the kit manufacturer's recommendation (Clontech Palo Alto, CA), the difference in mRNA levels needs to be at least 5 fold to allow reliable detection.

Here, we have developed a novel approach to the identification of differentially expressed rare sequences through a combination of subtractive hybridization and RNA amplification, termed a Subtractive Transcription-based Amplification of mRNA (STAR). In our method, the expressed RNAs from two source are used for the preparation of specialized cDNA libraries, from which single stranded (+) sense tester RNA and single stranded (-) sense driver DNA are generated. Subtraction is accomplished by the hybridization of single-stranded driver DNA to the complementary single-stranded tester RNA, followed by RNase H digestion. This process not only eliminates the necessity for physical removal of hybridized common sequence, but also eliminates the self-annealing step of the tester nucleic acids that is required by the SSH method for the amplification of target sequences. The self-annealing step usually imposes kinetic limitations and requires lengthy hybridization in order to recover rare cDNA sequences. Furthermore, in the STAR method, the tester RNA is designed with a defined terminal sequence which allows the unhybridized tester RNA to be amplified in a linear RNA amplification process, rather than exponential PCR amplification, thereby minimizing biased sequence amplification [12,13] Since the tester RNA remains unchanged in the process, the products of one round of STAR are used directly in subsequent rounds to provide further enrichment of the unique sequences.

In this study, we applied the STAR method to an investigation of changes in gene expression in port-mortem AD brain. We show that, indeed, the STAR process significantly increases the levels of unique and rare sequences relative to abundant housekeeping genes and identified changes in the expression of genes not previously linked to the AD. We also performed extensive literature mining to provide a basis for considering their likely involvement in neurodegenerative processes.

## Results

### Characterization of the subtracted AD cDNA libraries

**Figure 1:**
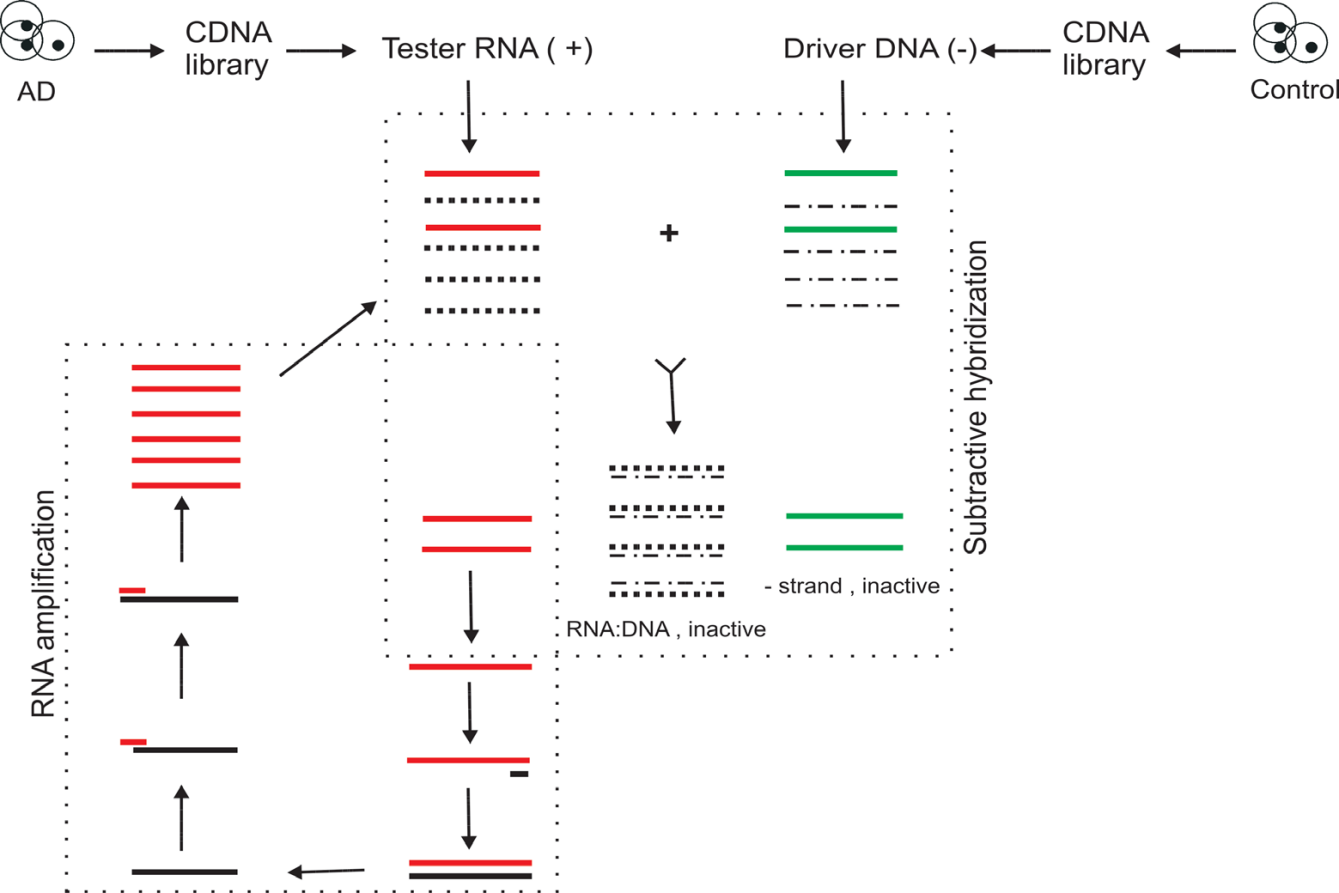
**Schematic diagram of the STAR procedure**. The solid red line represents tester specific RNA (+). The solid green line indicates driver specific DNA (-). The solid black line shows newly synthesized tester specific first strand cDNA (-). Short-dashed lines show the common tester RNA (+). The long and short mixed dashed line denotes the common driver DNA (-).

Two subtracted cDNA libraries, using either the 3' cDNA of the control sample (C-AD) or the 3' cDNA of the AD sample (AD-C) as tester, were prepared using the STAR method (Fig 1). It was expected that they would contain genes that were down regulated (C-AD) and up regulated (AD-C) in AD brain, respectively. To isolate these genes, three rounds of STAR subtraction were performed, and the remaining 3' biased cDNA tester fragments were linearly amplified, ligated into a pUC-modified vector and transformed into *E. Coli *DH10B cells. Approximately 5000 colonies were obtained for each STAR library. Subsequently, PCR and DNA sequencing were performed on amplified cDNA inserts from 600 individual colonies for each subtracted library. Data analysis indicated that this number of colonies was sufficient to represent the entire subtracted library since many genes, including some of low abundance, appeared to be represented by more than one colony. The results summarizing the genes found in the C-AD and AD-C libraries are listed in Tables 1 and 2, respectively.

Of the 600 colonies analyzed from each library, over 500 contained inserts and produced readable sequences after PCR. Sequence annotation grouped the genes into three categories: (i) known genes, (ii) ESTs and (iii) novel genes, whose sequences either only match a fragment of genomic DNA or the database search did not hit any existing known sequence. The known genes were represented by multiple colonies (Table 1, 183/266, i.e., out of 266 colonies sequenced from the C-AD library 183 were unique sequences of known genes; Table 2, 187/277 in the AD-C library) far more often than the novel genes (113/131 and 109/119), indicating that known genes are relatively more abundant than the novel genes, explaining why they are more easily identified by traditional approaches. Sequence annotation also revealed that genes involved in maintenance of neuronal cell structure and function, the ubiquitin pathway and energy metabolism were predominant in the C-AD library, indicating that they were down regulated in AD brains. Whereas in the AD-C library, genes involved in inflammation, protein translation, cytoskeleton/cell adhesion and apoptosis/neurodegeneration were up-regulated in AD. Over 55% of the sequenced fragments in each subtracted library represented unknown genes, of which one half were not previously described as cDNA sequences. Representative data from these libraries is shown below.

### Validation of changes from the subtracted libraries

More than 40 genes from the C-AD library were randomly selected for analysis by qRT-PCR using the pools of control and AD mRNAs as input. The results confirmed down-regulation of over 80% of them, consistent with the results of the subtracted library (Table 3). The data also confirmed that a majority of these genes (over 90%) were less abundant than β-actin. Furthermore, all novel transcripts were expressed at extremely low levels, some of them at only 0.0001–2% of the abundance of β-actin (Table 3). Taken together these results indicated that the STAR method permitted the isolation of differentially expressed genes, including very rare ones. This approach also revealed the existence of large number of previously unidentified genes expressed in the human brain which appear to have a disease association.

Nearly 40 randomly selected genes from the AD-C library were also analyzed by qRT-PCR in order to further validate the library data. In this case, less than 50% of the selected transcripts were found to be up regulated, with a lower percentage of novel genes (Table 4). This might be indicative of a higher content of false positive in this library, pointing out to the necessity of independent validation prior to gene selection for down stream functional studies. One possibility for the relatively low confirmation rate of the data from the AD-C library is that the increases in the expression of unconfirmed transcripts were small and, therefore, might also be difficult to validate by qRT-PCR, although this wasn't evident in the qRT-PCR validation of down regulated genes from the C-AD library. Despite this, the technique clearly identifies novel sequences that are up regulated in AD brain.

### Supporting evidence for gene association with AD

An additional approach to increase confidence in the identified genes as likely disease candidates was to mine knowledge pertaining to gene function in AD in the existing literature using a custom built literature mining tool, LitMiner. Using this approach, we generated short lists of genes whose association with AD could be further rationalized (Tables 5 and 6).

The data mining analysis identified 12 genes (Table 5 items 1–12), whose down-regulation in AD has been previously reported, in agreement with our results as these genes were present in the C-AD subtractive library. Furthermore, down-regulation of synuclein alpha was also confirmed by qRT-PCR analysis (Table 5, item 12). The remaining genes on this list (items 13–37) have never been formally linked to AD, but their possible involvement could be either inferred from the literature or from the results of the present study. For example, mutations in A2M, BRI3, EEF1A1, EIF2S2, MAPT and UBB (items 13–18) have been linked to various other neurodegenerative diseases [14-19] (Similarly, ATB2B1, CDK5R1, CPLX1, NES, RAD21, ST13 and TAGLN3 (items 19–25) have been shown to be down-regulated in some brain disorders [20-26] The down-regulation of 12 genes from the C-AD library (items 26–37) was validated by qRT-PCR (Table 3) and a similar literature analysis further supports the potential involvement of RELN, PRKCE and SYT4 (items 35–37) [2,27,28] Therefore, it is likely that the down-regulation and the loss of these gene functions could play a role in AD.

Table 6 contains a short list of genes cloned from AD-C subtractive library and supporting evidence for their role in AD. Sixteen of these genes (Table 6, items 1–16) have been previously associated with AD. For example, up-regulation of CLU, GFAP, MT1F (items 1, 2 and 10, respectively) have often been reported in AD brains [29-31] This is consistent with our data from the AD-C library. Two separate groups, Takahashi et al 2000 [32] and Loring et al 2001 [3], reported up-regulation of STXBP1 (item 16) in AD and the transcript of KNS2 (item 17) was reported elevated in injured optic nerve [33]. Both genes were found in the AD-C library and KNS2 was represented by multiple clones; however, we could not validate these changes by qRT-PCR (Table 5). Seven genes from this list (Table 6, items 17–23), have been shown to be elevated in other neurological disorders such as Schizophrenia, Down syndrome and ischemic neurodegeneration. Changes in the additional 17 genes (Table 6, items 24–40) were validated by qRT-PCR, six of which (items 35–40) could be also inferred from the literature.

**Figure 2:**
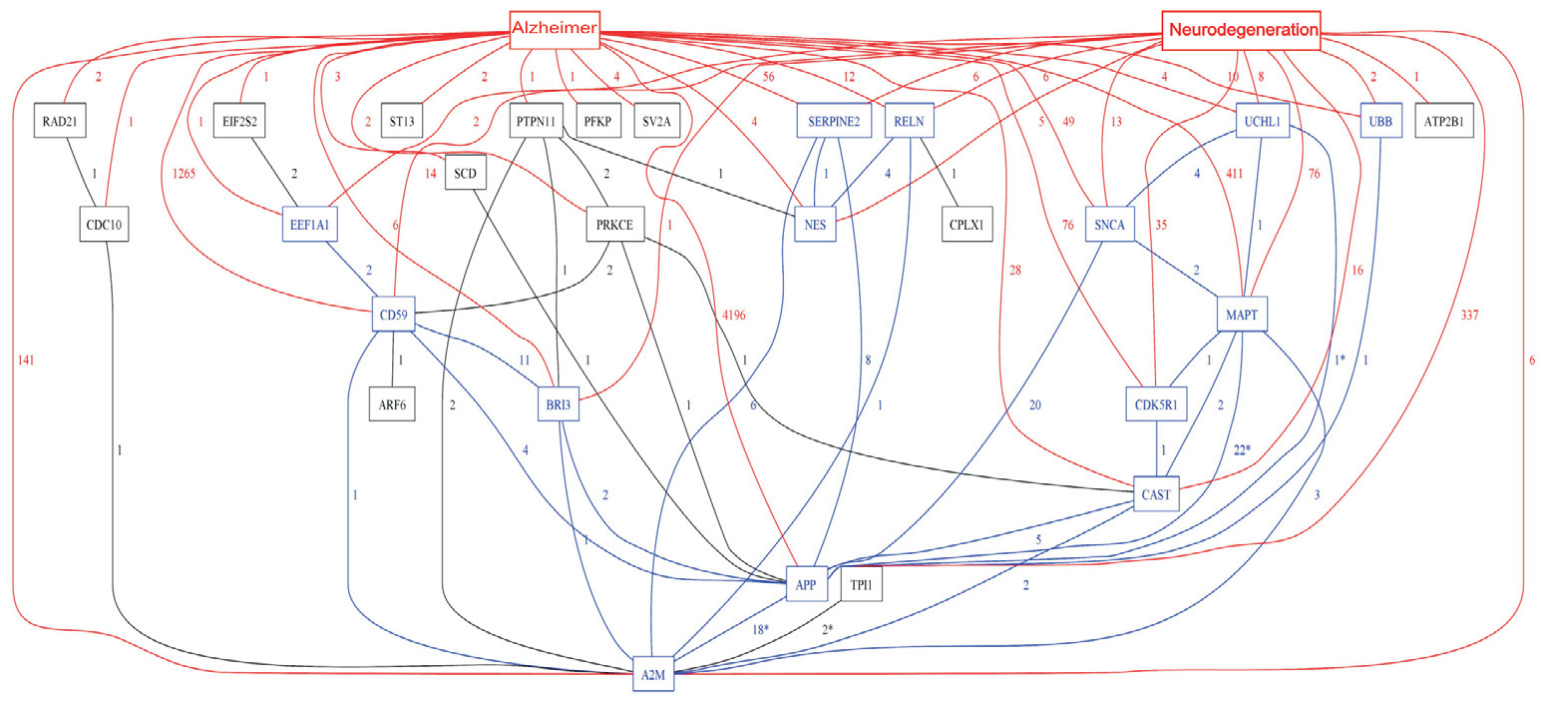
**Association of down regulated genes with AD and neurodegeneration**. The associations between genes or between genes and knowledge term (Alzheimer or Neurodegeneration) are represented as a graph with the frequency indicated by numbers on the links. A red box or line indicates a knowledge term or a link between a gene and a knowledge term, respectively. A blue box and line shows a gene that is associated with both knowledge terms. A black box and line denotes a gene that is associated with only one knowledge term or another gene.

**Figure 3:**
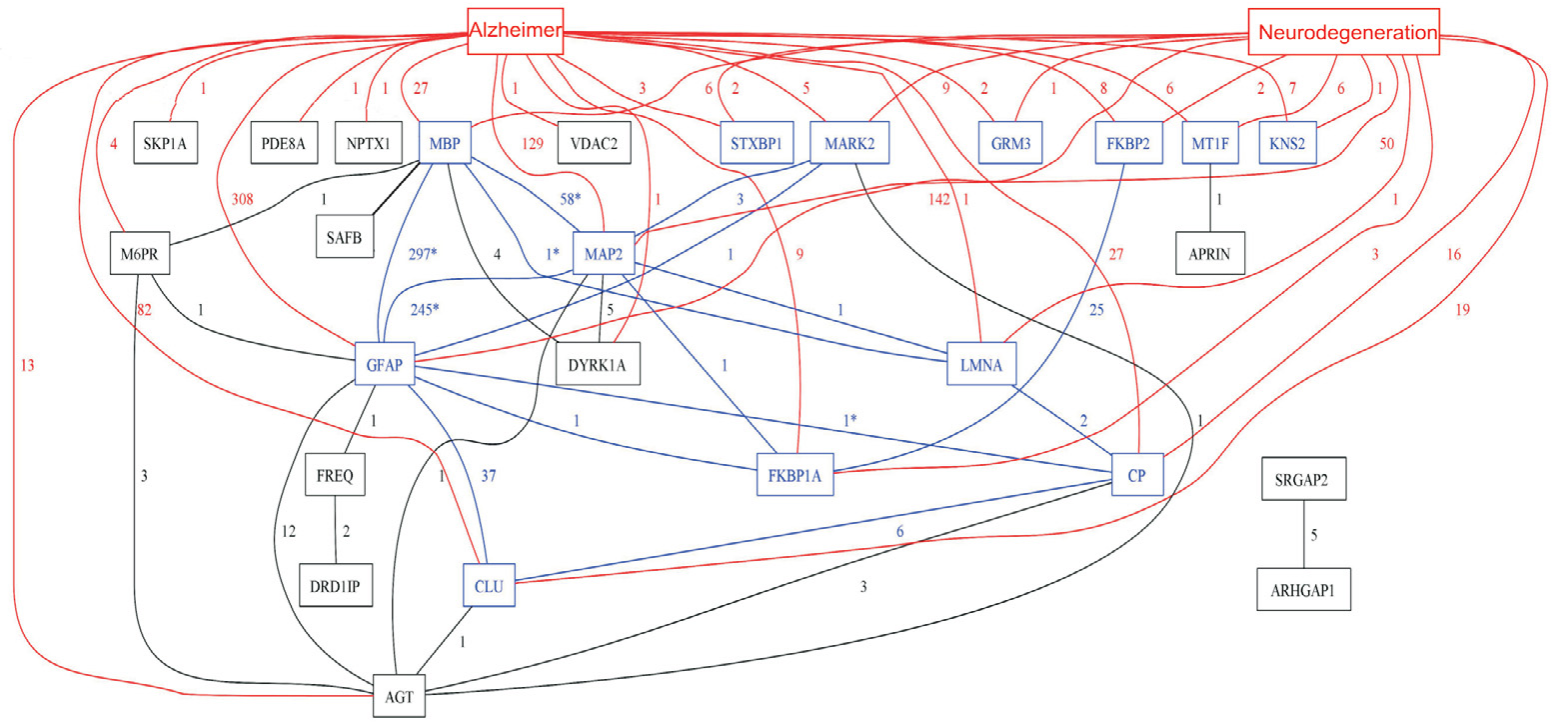
**Association of up regulated genes with AD and neurodegeneration**. The associations between genes or between genes and knowledge term (Alzheimer or Neurodegeneration) are represented as a graph with the frequency indicated by numbers on the links. A red box or line indicates a knowledge term or a link between a gene and a knowledge term, respectively. A blue box and line shows a gene that is associated with both knowledge terms. A black box and line denotes a gene that is associated with only one knowledge term or another gene.

Possible associations between the 37 down-regulated (Fig. 2) and 40 up-regulated (Fig. 3) genes from the subtracted libraries were mapped by additional literature mining. We imported the Unigene names of each group of genes into LitMiner and used "Alzheimer" and "neurodegeneration" as knowledge terms. All articles containing a gene name, or alias and the knowledge terms, in either title, MeSH terms or abstract were reported in a graphic format. Any two given genes or a gene and one of the knowledge terms appearing in the same article were considered to constitute an association. The frequencies of these associations are represented by the number adjacent to the connecting lines. Within LitMiner, we could rapidly retrieve and manually scan articles to eliminate false association due to the misuse of gene aliases in the database. The graphs in Figs 2 and 3 contain genes that exhibited actual association to the knowledge terms or to other genes. Some genes summarized in Table 5 and 6 are not included in Figs 2 and 3 because the current software finds only associations identified in the title, abstract or MeSH terms. Nevertheless, the LitMiner output gives a high level overview of the relationships of these differentially expressed genes that is not obtainable from a simple gene list.

## Discussion

With the advent of high throughput genomics and proteomics technologies, the involvement of new genes in AD continues to emerge, indicating clearly that the full extent of molecular and functional aberrations responsible for the etiology of AD is not yet understood. The current high throughput methods are mainly designed to identify changes in relatively abundant genes, whereas weakly expressed genes or proteins are still overlooked. For example, cDNA microarrays, although widely used, are limited to known mRNA sequences or ESTs, thus it is not suitable for the identification and isolation of novel rare transcripts. Complementary to microarray technology, subtractive hybridization is capable of detecting changes of both known and novel genes. The existing SSH method enriches the differentially expressed genes by selectively suppressing the amplification of non-differentially expressed transcripts in the PCR reaction, resulting in the enrichment of differentially expressed transcripts. An annealing step is necessary for PCR amplification and subsequent cloning and since rare sequences re-anneal more slowly, this process reduces their chances of discovery. The STAR technology uses single strand RNA/DNA hybridization to remove non-differentially expressed genes. Rare sequences are enriched by slower hybridization and the non-hybridized RNA is recovered and amplified by a linear RNA amplification procedure, which minimizes the biased exponential amplification of subsets of genes in a mixture. These features make the STAR method attractive for examining changes of gene expression in normal and diseased brain tissues, where many mRNAs are present in low abundance. In the present study, we used the STAR method to identify genes that are not only novel in human brains, but also differentially expressed in AD. Our subtracted cDNA libraries constructed by the STAR technology contained genes whose relative differences in expression levels ranged from 25% to 100%, illustrating the fidelity of this method. Although there has been little, and in some cases, no overlap in identified genes between different microarray studies of AD samples, due to experimental variability, different sources of microarray bearing different sets of genes and different areas of brain tissues used, STAR has identified a number of known, moderately abundant genes, whose alternation in AD was in agreement with previous microarray analyses. Of these APP and SNCA were found down regulated [2], AGT [34], CLU [34], FTL [3,34], MAP2 and STXBP1 [3] were reported up-regulated. More than 55% of clones in each subtracted library contain cDNA of unknown function and more then 25% are novel sequences only matching genomic DNA in database searches. These novel genes can now be vigorously pursued to identify their function and precise role in AD pathology.

Similar to other high throughput approaches, the changes in gene expression identified by the STAR method need to be validated by qRT-PCR. This is especially true for the rare and novel sequences, for which there is no information available in the literature. Combinations of PCR-selected cDNA subtraction and cDNA microarray analysis of the subtracted clones, or using probes generated by PCR-selected subtraction to screen Affymetrix GeneChips have been attempted [35,36]. The clones produced by STAR can be printed on microarray slides for hybridization with labeled cDNAs from individual brains samples, however, the rare RNA species must be enriched by amplification in order to give reliable hybridization signals. This method, currently under development, could be a high throughput approach to eliminate false positives before qRT-PCR validation on individual genes. We have measured changes in nearly 40 genes from each subtracted cDNA library by qRT-PCR. However, there was a difference in the confirmation rate between the two subtracted libraries. Besides the fact that there seemed to be more down- than up- regulated genes in the AD brains where neurodegeneration was already evident [2,3]., we cannot offer an definite explanation why some genes, such as, Syntaxin binding protein 1 and kinesin 2 found up-regulated by STAR procedure, consistent with the results obtained by others [3,32,33], yet not confirmed by qRT-PCR. Further investigations with different fine-tuned qRT-PCR primers and conditions and by using individual brain samples may offer insight on this issue.

While identification of novel genes functions in neurodegeneration remains to be our ongoing objective, the current study was focused on the association of some of the known genes with AD. Among the down regulated genes in Table 5, those belonging to two major functional categories are noteworthy. The first group contains genes involved in maintenance of neuronal cell function, including CAST, PFRK, SERPINE2, SNCA, SV2A and SYT4 [2,37-41] Their roles in normal brain are to promote neurite outgrowth and to regulate synaptic vesicle transport or trafficking at the synapse. Their down regulation is consistent with the compromised synaptic transmission observed in AD brain. This undoubtedly contributes to the impairment of memory and cognitive function. Our findings support the current view that AD is a disease of synaptic failure [42].

The second group of genes consists of members of the ubiquitin pathway involved in protein degradation. Although only UBB, UBE2B and UCHL1 passed our current screening criteria, several other ubiquitin pathway-related genes also appeared in the C-AD library. A straightforward interpretation of this result would be that the decreased ability of protein degradation caused by the down regulation of ubiquitin pathway genes, resulted in the accumulation of unwanted proteins in the senile plaques and neurofibrillary tangles. However, studies of individual ubiquitin pathway genes, such as UCHL1, suggest other mechanisms. For example, UCHL1 is sensitive to redox changes and is oxidized in AD brain [43,44]. indicating that its function under stressed conditions is more than "house keeping" [45]. Elevated expression of other ubiquitin pathway proteins in response to oxidative stress has also been documented. [46,47] However, a better understanding of the role of individual ubiquitin pathway genes in AD pathology requires comprehensive study.

Extensive evidence suggests that inflammation plays a major role in AD [48]. It is therefore not surprising to find genes, such as MBP, NPTX1 and SCARB2 involved in neuroinflammation [49-51], to be up regulated in AD brains (Table 6). We also found several genes related to the cellular distribution of iron (FTL), transport of copper (CP) and metal-binding (MT1F) that were up-regulated in AD (Table 6). [31,52,53] These data strongly suggest a disruption of metal homeostasis and a potential metal neurotoxicity component in AD. The increase of these proteins may indicate an acute phase-type reaction and/or a compensatory response to stress conditions.

We did not find any typical cytoskeletal proteins, such as members of the actin or tubulin families, to be significantly up-regulated in AD brain. However, we did identify three clusters of genes related to cytoskeletal organization (Table 6). The first group contains organelle membrane skeletal proteins such as SAFA, LMNA and SPTBN2 [54-56] The second group encodes skeletal binding proteins, including FKBP2, interacting with erythrocyte membrane cytoskeletal protein [57], KNS2 and MAP2, all associated with microtubules [58,59] The third group contains GTPase activating proteins, ARHGAP1, CENTG2 (AGAP1), SRGAP2, which regulate membrane trafficking and actin remodeling [60-62] SEPT6 can also be categorized into this group since it is a polymerizing GTPase required for cytokinesis and cortical organization [63]. It is intriguing that so many cytoskeleton related genes are up regulated in the degenerating brains. It is currently unclear whether these changes were the causes or the consequences of neurodegeneration. One possibility is that these genes might be up regulated in activated microglia or astrocytes, which triggered signals that mediate cytoskeletal reorganization and vesicular trafficking during glial cell migration.

## Conclusion

We have used a proprietary subtractive hybridization technology (STAR) to identify differentially expressed genes in AD brains, extending existing gene profiling and subtraction methods, such as DNA or protein microarray analyses to identify rare sequences. 55% of the identified differentially expressed genes have no known function, of which, 25% had no matching ESTs in the databases. These sequences represented novel and newly discovered transcripts in the brain and were also differentially expressed in AD brains. Using literature mining tools we have established (Figs. 2 and 3) many new gene associations, not yet reported to be involved in AD. This information will facilitate future efforts aimed at establishing alterations in molecular pathways involved AD pathology.

## Methods

### Brain tissues and RNA extraction

Poly A+ RNA was isolated from the frontal cortex of frozen post mortem human brains from the same 4 AD and 5 age-matched control subjects as used in our previous study using the same the extraction procedure [34]. Equal amounts of mRNA were taken from each brain and subsequently combined to generate separate pools of AD RNA and normal RNA. Five micrograms of the mixed mRNA from each pool was converted to double-stranded cDNA (ds-cDNA) and used to prepare full-length tester and driver cDNA libraries.

### Subtractive Transcription-based Amplification of mRNA (STAR)

The STAR subtraction procedure was performed as illustrated in Figure 1. Briefly, single stranded (+) sense tester RNA and single stranded (-) sense driver DNA were generated from specialized tester and driver cDNA libraries (see below), respectively. Subtraction was accomplished by hybridization of single-stranded driver cDNA to the complementary single-stranded tester RNA, followed by RNase H digestion. The unhybridized tester RNA remained active when subjected to a linear RNA amplification process comprising the steps of (i) reverse transcription, to synthesize cDNA from the tester RNA; (ii) DNA conversion, to append a promoter to the cDNA; and (iii) *in vitro *transcription, to synthesize additional copies of tester RNA.

#### Construction of full length tester and driver cDNA libraries

Double-stranded cDNA (ds-cDNA) was synthesized from 5 μg of mRNA from each pool of brains using a modified ThermoScript™ ds-cDNA synthesis kit (Invitrogen, Burlington, ON) and a locking-dT_19_V oligonucleotide comprising a Not I restriction enzyme site. An Asc I adaptor was then ligated to the 5' terminus of the ds-cDNA. Following digestion with Asc I and Not I enzymes (NEB, Pickering, ON), the ds-cDNA was directionally ligated into pUC18-derived vectors, p17^+ ^for the production of driver DNA and p14 for the production of tester RNA. Both vectors contained a T7 promoter and specific oligonucleotide sequences (OGS302: 5'-GCCTGCACCAACAGTTAACA, in the case of p17^+^, and OGS77: 5'-CGAGAGCACCTGGATAGGTT, in the case of p14), immediately upstream of the cDNA inserts. These plasmids were then transformed into *E. coli *DH10B cells to generate the full length cDNA libraries.

#### Construction of 3'-UTR tester and driver cDNA libraries

Subtraction using STAR was performed using only the more variable 3'-UTR regions of mRNA sequences in order to minimize losses of gene family members that share homologous 5'-UTR and coding regions. Thus, 3'-UTR tester and driver libraries were subcloned from the original full-length p14 and p17^+ ^cDNA libraries as follows. Plasmid DNA (2 μg) from each library was digested with Not I restriction enzyme and purified using Qiaquick (Qiagen, Mississauga, ON) and 1 μg of each was used to *in vitro *transcribe (IVT) full-length RNA copies of the cDNA inserts with T7 RNA polymerase, according to the manufacturer's instruction (USB, Cleveland, OH). The plasmid DNA template was digested with 2U RNase-free DNase I (Promega, Madison WI) and the RNA was purified with RNeasy kit (Qiagen). The newly synthesized RNA from each library now contained specific oligonucleotide sequences OGS77 and OGS302 at its 5' terminus, which were initially carried by p14 and p17^+ ^plasmid vectors, respectively. Twenty micrograms of each IVT RNA were converted to first-strand cDNA (as described above) and purified by Qiaquick (Qiagen). Second-strand cDNA synthesis was then accomplished in a reaction containing Klenow DNA polymerase and specific oligonucleotide primers, OGS77 or OGS302, according to the manufacturer's instruction (NEB, Pickering, ON). The resulting full-length ds-DNA for each library was purified by Qiaquick (Qiagen). To prepare the 3'-UTR tester and driver libraries for STAR, 6 μg of the full-length ds-DNA for each library was divided into 1 μg aliquots and each aliquot was digested with one of six restriction enzymes (Bsh 1236 I, HinP1 I, Mse I, Msp I, Rsa I or Sau3A I) (NEB and MBI Fermentas, Burlington, ON). Following digestion, each set of 6 reactions was extracted with phenol, pooled and desalted. Each pooled DNA sample was blunt-ended using T4 DNA polymerase (NEB) and ligated to 2.5 μg Asc I linker (NEB) in a 10 μL reaction. Each linker-adapted DNA sample was then digested with Asc I and Not I enzymes (NEB) and purified using Qiaquick (Qiagen). The digested DNA samples were then ligated into *Asc *I-*Not *I digested p14 and p17^+ ^plasmid vectors respectively and transformed into *E. coli *DH10B cells. The resulting transformants for each library were pooled to produce the 3'-UTR p14-tester and p17-driver libraries.

#### Construction of 3' STAR subtracted cDNA libraries

One microgram of plasmid DNA isolated from each 3'-UTR library was digested with Not I and purified by Qiaquick (Qiagen), and then *in vitro *transcribed as described above to produce RNA. The RNA copies from the tester library are now ready to be used in STAR. Twenty micrograms of the p17-3' driver RNA were further converted to single-stranded driver DNA in a first-strand cDNA synthesis reaction as described above with the exception that a oligo rU, instead of oligo dT, was used as primer for the cDNA synthesis and the rU primer attached to every first strand cDNA was then digested with RNase A. The p14-3' tester RNA (10 ng) was then hybridized with 100-fold excess p17-3' single-stranded driver DNA in a hybridization buffer containing 40 mM Tris-HCl, pH7.5, 0.1 M NaCl, 7.6% D-Trehalose and 40% DMSO. The reaction was carried out in a thermocycler in descending temperature sequence as follows: 65°C, 63°C and 61°C each for 10 min; 59°C for 30 min; 57°C, 55°C, 53°C, 51°C, 49°C, 47°C and 44.5°C each for 98 min; 42.5°C for 18 hours, followed by RNase H digestion at 40°C for 30 min. The tester RNA was then converted to cDNA and amplified by in vitro transcription as described above. After 3-rounds of STAR (Fig. 1), the remaining tester RNA was converted to double-stranded DNA, digested with *Asc *I and *Not *I and ligated into a similarly digested pUC-modified vector. The plasmids containing specific tester DNA inserts were transformed into *E. coli *DH10B cells to form the STAR libraries.

### Analysis of STAR cDNA libraries from human brain samples

A STAR library, where AD 3' cDNA was used as tester (termed the AD-C library) which should contain genes up-regulated in AD brains. Conversely, when control 3' cDNA was used as tester, we produced a C-AD library, which should permit the isolation of genes down regulated in AD. Approximately 600 individual colonies from each subtracted library were picked and the cloned inserts were amplified by PCR with HotStart Taq polymerase (Qiagen) using forward and reverse flanking primers on the vector. The PCR amplicons were purified using the Corning filter polystyrene 96-well plate system (Fisher Scientific, Ottawa, ON). One microliter of the purified PCR product was used for sequencing on the ABI Prism 377 DNA sequencer or 3100 Genetic Analyzer. DNA sequences were analyzed using Sequencher and batch BLAST search.

### cDNA synthesis, and qRT-PCR

cDNA was synthesized from the same RNA pools used to construct the original AD and control cDNA libraries using Superscript II Reverse Transcriptase according to the manufacturer's instruction (Invitrogen). The reaction was stopped by adding EDTA to a final concentration of 5 mM. RNA templates were subsequently hydrolyzed in 0.5 M NaOH solution at 65°C for 20 min. The cDNA was further purified using a QIAquick PCR purification kit (Qiagen) and quantified using the OliGreen ssDNA Quantitation Kit (Molecular Probe, Hornby, ON). Forward and reverse primers for sequences of interest were designed using Primer Express (Applied Biosystems, Foster City, CA). Equal amounts of cDNA (2 ng each) were used for qRT-PCR analysis using the QuantiTect SYBR Green PCR Kit (Qiagen) according to the manufacturer's instructions. Fluorescent products were detected using a GeneAmp 5700 Sequence Detection System (PE Applied Biosystems). Percentage of changes was calculated according to the manufacturer's instruction. The experiments were performed in triplicate. Only significant differences (ρ < 0.05; t-test on the qRT-PCR experiments) between AD and control samples are reported as differentially expressed genes.

### Literature mining

UniGene symbols of the known genes from each subtracted library were obtained from the SOURCE database using their respective Genbank accession numbers. These symbols were imported into a literature mining prototype software, LitMiner [64], developed by the National Research Council of Canada to identify relationships among genes and their association with biological processes. The search uses the standard UniGene symbols and all possible aliases appearing in the title, abstract or MeSH terms of publications. The associations amongst genes are represented as a graph with the frequency indicated by numbers on the links. Occasionally, the numbers in the graphs may not accurately represent associations because some articles might simply mention genes or terms without specifying an association or using incorrect or incomplete gene aliases. In practice these errors are manageable, because within LitMiner, we can rapidly retrieve and manually scan articles to eliminate such false associations. A knowledge term, such as "Alzheimer" or "Neurodegeneration" was added into the search to explore possible association of these genes with AD. The LitMiner tool was used since it is a much faster than searching for gene relationships manually. In this context, relationship means a co occurrence between either gene names or gene names and a biological process.

Every step performed with LitMiner for this study could be replicated manually using Entrez Gene, PubMed, and graph drawing tools available in Microsoft Word or similar sources. The first step of this manual process would be to find all the gene name aliases available in Entrez Gene for each known UniGene symbol from each subtracted library. Second, each such list of aliases would be converted into a disjunctive PubMed query by adding the OR operator (|) after each alias. As well, any alias that is longer than a single word would be enclosed in quotation signs (" "). For example, part of the query for the gene A2M would be, "*A2M" | "alpha 2 macroglobulin" | "alpha2 M*". Other manual improvements could be made to this query, i.e., to remove overly general terms. Third, researchers would scan the results removing incorrect or uninformative matches. Fourth, a database or file would record all the PMIDs that matched each of these gene queries in PubMed. Fifth, queries would be created for biological processes and the resulting lists of PMIDs would also be included in the database. Using this database, the researcher could then count how many PMIDs were shared by any pair of genes or biological process. For example, there would be tens of PMIDs whose title, abstract, and MeSH terms mention both A2M and APP. Once all co occurrence counts are collected, those counts could be entered into a manually drawn graph. These steps would require a considerable amount of time, to remove incorrect matches and to produce graphs of the co occurrences between gene names and biological processes.

## Authors' contributions

QYL participated in the design of the study, analysis and interpretation of the data and preparation of the manuscript. RRS performed the STAR experiments and helped to analyze the data and draft the manuscript. LTM invented the STAR procedure and contributed to the interpretation of the data. MRL constructed the regular cDNA libraries, performed the qRT-PCR analysis. JXL and HS both participated in the qRT-PCR analysis and the interpretation of the data. BL provided comprehensively classified postmortem human control and AD brain tissues. JM developed LitMiner software and contributed to writing of the manuscript. PRW and MS both participated in the design of the study and contributed to writing of the manuscript. All authors read and approved the final manuscript.

## Figures and Tables

**Table 1:** Summary of sequenced clones from the C-AD subtracted cDNA library

Sequence type	Number of colonies sequenced	Number of unique genes	Library representation (%)
Known	266	183	44.7

Unknown:	261	226	55.3
• ESTs	131	113	27.6
• Genomic DNA or no hit	130	113	27.6

Total	527	409	100.0

**Table 2:** Summary of sequenced clones from the AD-C subtracted cDNA library

Sequence type	Number of colonies sequenced	Number of unique genes	Library representation (%)
Known	267	187	44.2

Unknown	261	236	55.8
• ESTs	142	127	30.0
• Genomic DNA or no hit	119	109	25.8

Total	528	423	100.0

**Table 3:** Summary of qRT-PCR analysis of the selected genes from the C-AD library

**Name**	**Acc #**	**Gene Description**	**% Down**	Abundance relative to β-actin*
	AF498312	integrin-linked kinase1 (ILK)	29%	19.00%
ANK2	NM_020977	ankyrin2, transcript varient 2	no change	11.00%
GABRB3	CR749803	Gamma-aminobutyric acid A receptor, beta 3	no change	98.00%
UBE2B	NM_003337	ubiquitin-conjugating enzyme E2B	45%	33.00%
	AY339422	NADH dehydrogenase sububit 3	30%	475.00%
TPI1	BC017917	triosephosphate isomerase	41%	208.00%
BCAS1	NM_003657	breast carcinoma sequence 1	20%	71.00%
E46L	AK095309	Ataxin-10	no change	58.00%
SCA1	NM_000332	spinocerebellar ataxia 1	68%	3.00%
DEAF1	BC053322	transcriptional regulator protein, suppressin	24%	7.00%
HLF	M95585	hepatic leukemia factor(HLF)	no change	0.70%
CKIP-1	BC010149	TNF intracellular domain-interacting protein	35%	2.00%
SNCA	NM_000345	synuclein, alpha SNCA	68%	0.20%
CRI1	AL357456	CREBP/EP300 inhibitory protein 1or RBP21	19%	170.00%
SYT4	BC036538	synaptotagmin IV	58%	74.60%
ARF6	BC030291	ADP-ribosylation factor 6, Arp6	59%	0.82%
PRKCE	BC054052	protein kinase C, epsilonY	15%	16.90%
	BC000143	ELMO2 engulfment and cell motility 2	55%	0.07%
SV2A	BC045111	synaptic vesicle glycoprotein 2A	19%	2.00%
RELN	NM_005045	reelin	82%	1.20%
	BC042904	hypothetical protein DKFZp564A176	15%	22.20%
	NM_152722	hypothetical protein FLJ25530	no change	225.00%
	AK056024	EST	49%	10.60%
	DR005162	EST	21%	1163.00%
	AL162511	genomic DNA	49%	26.00%
	AC096861	genomic DNA	19%	16.00%
	AC145098	genomic DNA	41%	12.00%
	AL590482	genomic DNA	86%	0.150%
	AL121908	genomic DNA	18%	0.0050%
	AL513318	genomic DNA	47%	0.0002%
	AC099787	genomic DNA	100%	0.0012%
	AL354884	genomic DNA	100%	0.0012%
	AC119800	genomic DNA	50%	0.0003%
	AC092631	genomic DNA	40%	0.0002%
	AL138753	genomic DNA	74%	0.0002%
	AL356608	genomic DNA	76%	0.0020%
	AC106856	genomic DNA	17%	0.0001%
	AC012071	genomic DNA	no change	0.0290%
	AC112717	genomic DNA	39%	0.0019%
	AP002803	genomic DNA	64%	0.0140%
	AL158212	genomic DNA	no change	0.0002%

* Abundance relative to β-actin was calculated using a relative quantitation formula : 2^-ΔCt ^x%, where ΔCt is the qRT-PCR cycle number of target gene minus the qRT-PCR cycle number of β-actin at threshold in the same round of qRT-PCR experiment using cDNA from control brains.

**Table 4:** Summary of qRT-PCR analysis of the selected clones from the AD-C library

**Name**	**Acce #**	**Description of matched genes**	**% Up**	**Abundance relative to β-actin**
USP10	NM_005153	ubiquitin specific protease 10	38%	12.50%
FNBP2	XM_059095	formin binding protein 2	19%	1.10%
LMNA	NM_170708	lamin A/C	36%	6.0%
RPS20	NM_001023	ribosomal protein S20	23%	12%
PREPL	AB007896	prolyl endopeptidase-like	25%	21.90%
SKP1A	NM_170679	cyclin A/CDK2-associated protein p19	41%	67%
MARK2	BC008771	ELKL motif kinase	32%	0.84%
AHCTF1	AB059277	AT hook containing transcription factor 1	60%	10.90%
PLRG1	BC020786	pleiotropic regulator 1	32%	0.34%
APRIN	NM_015032	androgen-induced proliferation inhibitor	32%	19.80%
CENTG2	NM_014914	centaurin, gamma 2	41%	6.60%
SCARB2	NM_005506	scavenger receptor class B, member 2	42%	416%
SPTBN2	NM_006946	spectrin, beta, non-erythrocytic 2	47%	34.50%
SEPT6	NM_015129	septin 6	27%	59%
SAFB	NM_002967	scaffold attachment factor B	42%	7.10%
DRD1IP	NM_015722	dopamine receptor D1 interacting protein	40%	26%
ARHGAP1	NM_004308	Rho GTPase activating protein 1	26%	241%
KNS2	NM_005552	kinesin 2 60/70kDa, transcript variant 1	no change	4.10%
KIAA1078	NM_203459	calmodulin regulated spectrin-associated protein 1-like 1	no change	25%
ANKMY2	BC035353	ankyrin repeat and MYND domain containing 2	no change	17%
FBXO33	BC053537	F-box protein 33, mRNA	no change	0.78%
KLHDC2	NM_014315	kelch domain containing 2	no change	100%
RPRC1	NM_018067	arginine/proline rich coiled-coil 1	no change	28%
HSPH1	NM_006644	heat shock 105kD	no change	48%
C21orf33	NM_004649	chromosome 21 open reading frame 33	no change	48%
MBD1	AF120981	methyl-CpG binding domain protein 1	no change	0.49%
STXBP1	NM_003165	syntaxin binding protein 1	no change	93.00%
NEK1	AF155113	NY-REN-55 antigen	no change	12.30%
ASB1	NM_016114	ankyrin repeat and SOCS box-containing 1	no change	9.10%
CCNI	NM_006835	cyclin I	no change	49%
	AL109709	EST	no change	22%
	BC035832	EST	17%	521%
	AP002833	genomic DNA	no change	6.30%
	AC104964	genomic DNA	no change	33%
	AP002833	genomic DNA	35%	0.06%
	AL138718	genomic DNA	50%	0.0032%
	AC091609	genomic DNA	no change	0.01%
	AL953889	genomic DNA	no change	1.60%

**Table 5:** A short list of down regulated genes and supporting evidence for their involvement in AD

**Item**	**Name**	**Acce #**	**Gene Description**	**Evidence for Down Regulation**
1	APP	BC065529	amyloid beta (A4) precursor protein	literature, [2], Down regulation in DS, [65]
2	CAST	NM_015576	CAZ-associated structural protein	literature, [40]
3	CD59	BC001506	CD59 antigen p18-20	literature, [66]
4	CDC10	BX648365	cell division cycle 10	literature, [67]
5	HK1	NM_033500	hexokinase 1	literature, [68]
6	PFKP	BC002536	phosphofructokinase	literature, [37]
7	PTPN11	NM_002834	protein tyrosine phosphatase, non-receptor type 11	literature, [69]
8	RPIP8	BC013240	RaP2interacting protein 8	literature, [3]
9	SCD	NM_005063	stearoyl-CoA desaturase	literature, [70]
10	SERPINE2	NM_006216	serine (or cysteine) proteinase inhibitor, clade E, member 2	literature, [41]
11	UCHL1	BC000332	ubiquitin thiolesterase, UCHL1	literature [71,72].
12	SNCA	NM_000345	synuclein, alpha	qRT-PCR, literature, [2]
13	A2M	BC040071	alpha-2-macroglobulin	mutation of A2M is linked to AD, [16]
14	BRI3	BC018737	Brain protein I3	mutation of BRI2 is liked to neurodegeneration[19]
15	EEF1A1	BC009875	Eukaryotic translation elongation factor 1 alpha 1	mutation, mutant mice exhibit neurodegeneration, [14]
16	EIF2S2	BC000461	Eukaryotic translation initiation factor 2, subunit 2 beta	mutation, mutations causing childhood ataxia, [15]
17	MAPT	BC032572	microtubule-associated protein tau, long splice form	mutation, mutation in exon 9–13 cause neurodegenerative diseases including AD, [18]
18	UBB	BC038999	ubiquitin B	mutation, frame shift mutation fund in AD, [17]
19	ATP2B1	AK024895	ATPase, Ca++ transporting, plasma membrane 1	inferred, expression is repressed by stress, [21]
20	CDK5R1	BC035448	cyclin-dependent kinase 5, regulatory subunit 1	inferred, [73], and in ethanol induced neurodegeneration, [23]
21	CPLX1	BC002471	complexin 1	inferred, Down regulated in schizophrenia, [24]
22	NES	AF086454	nestin	inferred, decreased in injured aged hippocampus, [20]
23	RAD21	NM_006265	RAD21 homolog	inferred, Down regulated by hypoxia, [26]
24	ST13	BC052982	suppression of tumorigenicity 13	inferred, rescue phosphorylated tau-induced cell death [25]
25	TAGLN3	AF303058	transgelin 3	inferred, down regulated in schizophrenia, [22]
26	ARF6	BC030291	ADP-ribosylation factor 6	qRT-PCR
27	BCAS1	NM_003657	Breast carcinoma amplified sequence 1	qRT-PCR
28	CKIP-1	BC010149	CK2 interacting protein 1	qRT-PCR
29	CRI1	AL357456	CREBP/EP300 inhibitory protein 1	qRT-PCR
30	DEAF1	BC053322	deformed epidermal autoregulatory factor 1	qRT-PCR
31	SCA1	NM_000332	spinocerebellar ataxia 1	qRT-PCR
32	SV2A	BC045111	synaptic vesicle glycoprotein 2A	qRT-PCR
33	TPI1	BC017917	triosephosphate isomerase	qRT-PCR
34	UBE2B	NM_003337	ubiquitin-conjugating enzyme E2B	qRT-PCR
35	RELN	NM_005045	reelin	qRT-PCR and inferred, down regulation or mutation cause autistic disorder, [27]
36	PRKCE	BC054052	protein kinase C, epsilonY	qRT-PCR and inferred, suppresses Aβ production, [28]
37	SYT4	BC036538	synaptotagmin IV	qRT-PCR, inferred, SYT(unspecified isoform) is down regulated in CA1 of AD brains, [2]

**Table 6:** A short list of up regulated genes and supporting evidence for their involvement in AD

**Item**	**Name**	**Acce #**	**Gene Description**	**Evidence for Down Regulation**
1	CLU	NM_001831	clusterin	literature, [30,34]
2	GFAP	NM_002055	glial fibrillary acidic protein	literature, [29]
3	CP	NM_000096	ceruloplasmin	literature, [53]
4	DYRK1A	NM_001396	dual-specificity tyrosine-(Y) phosphorylationregulated kinase 1A	literature, [74]
5	FKBP1A	NM_000801	FK506 binding protein 1A	literature, [57]
6	FTL	BC058820	ferritin, light polypeptide	literature [3,34,52]
7	M6PR	NM_002355	mannose-6-phosphate receptor	literature, [75]
8	MAP2	NM_031846	microtubule-associated protein 2	literature, [3]
9	MBP	BC008749	myelin basic protein	literature, [49]
10	MT1F	NM_005949	metallothionein 1F	literature, [31]
11	RPL23	BC010114	ribosomal protein L23	literature, [3]
12	RPS27	NM_001030	ribosomal protein S27	literature, [76]
13	VDAC2	NM_003375	voltage-dependent anion channel 2	literature, [77]
14	YWHAE	BC001440	tyrosine 3-monooxygenase/tryptophan-5-monooxygenase activation protein, epsilon polypeptide	literature, [78]
15	AGT	NM_000029	angiotensinogen	literature, [34,79]
16	STXBP1	NM_003165	syntaxin binding protein 1	literature, [3,32], qRT-PCR did not show significant change
17	KNS2	BC008881	kinesin 2 60/70kDa	inferred, up regulated in injured optic nerve, [33], qRT-PCR did not show significant change
18	C21orf33	NM_004649	chromosome 21 open reading frame 33	inferred, elevated in fetal Down syndrome brain, [80]
19	FKBP2	NM_057092	FK506 binding protein 2	inferred, [57]
20	FREQ	NM_014286	frequenin homolog	inferred, up in Schizophrenia, [81]
21	GRM3	NM_000840	glutamate receptor, metabotropic 3	inferred, GRM2 and 4 increased in ischemic neurodegeneration, [82]
22	NPTX1	NM_002522	neuronal pentraxin I	inferred, short pentraxins are upregulated in AD, [50]
23	PDE8A	NM_002605	phosphodiesterase 8A	inferred, family member of PDE8B, is upregulated in AD, [83]
24	AHCTF1	AB059277	AT hook containing transcription factor 1	qRT-PCR
25	APRIN	NM_015032	androgen-induced proliferation inhibitor	qRT-PCR
26	CENTG2	NM_014914	centaurin, gamma 2	qRT-PCR
27	LMNA	NM_170708	lamin A/C	qRT-PCR
28	PLRG1	BC020786	pleiotropic regulator 1	qRT-PCR
29	PREPL	AB007896	prolyl endopeptidase-like	qRT-PCR
30	RPS20	NM_001023	ribosomal protein S20	qRT-PCR
31	SAFB	NM_002967	scaffold attachment factor B	qRT-PCR
32	SPTBN2	NM_006946	spectrin, beta, non-erythrocytic 2	qRT-PCR
33	SRGAP2	XM_059095	SLIT-ROBO Rho GTPase activating protein 2	qRT-PCR
34	USP10	NM_005153	ubiquitin specific protease 10	qRT-PCR
35	ARHGAP1	NM_004308	Rho GTPase activating protein 1 (CDC42GAP)	qRT-PCR, inferred, CDC42 is upregulated in AD, [60]
36	DRD1IP	NM_015722	dopamine receptor D1 interacting protein	qRT-PCR, inferred, up regulated in schizophrenia, [84]
37	MARK2	BC008771	MAP/microtubule affinity-regulating kinase 2	qRT-PCR, inferred, [85]
38	SCARB2	NM_005506	scavenger receptor class B, member 2, CD36 antigen	qRT-PCR, Inferred, inflammation increases CD36 mRNA, thus induceCD36 antigen, [51]
39	SEPT6	NM_015129	septin 6	qRT-PCR, inferred, increased in motor deficient mice,[86]
40	SKP1A	NM_170679	S-phase kinase-associated protein 1A	qRT-PCR, inferred, overexpression of SKP1A together with APP increase the production of Aβ, [87]
